# Comparison of two different types of railway ballast in compression and direct shear tests: experimental results and DEM model validation

**DOI:** 10.1007/s10035-018-0843-9

**Published:** 2018-09-29

**Authors:** Bettina Suhr, Stefan Marschnig, Klaus Six

**Affiliations:** 1 Virtual Vehicle Research Center, Inffeldgasse 21/A, 8010 Graz, Austria; 2 Institute of Railway Engineering and Transport Economy, Graz University of Technology, Rechbauerstraße 12/II, 8010 Graz, Austria

**Keywords:** DEM simulation, Railway ballast, Model validation, Particle contact modelling

## Abstract

Railway ballast is an angular and coarse material, which demands careful DEM modelling and validation. Particle shape is often modelled in high accuracy, thus leading to computational expensive DEM models. Whether this effort will increase the DEM model’s overall prediction quality will also vitally depend on the used contact law and the validation process. In general, a DEM model validated using different types of principal experiments can be considered more trustworthy in simulating other load cases. Here, two types of railway ballast are compared and DEM model validation is conducted. Calcite and Kieselkalk are investigated under compression and direct shear test. All experimental data will be made openly accessible to promote further research on this topic. In the experiments, the behaviour of Calcite and Kieselkalk is surprisingly similar in the direct shear test, while clear differences can be seen in the stiffnesses in the compression test. In DEM modelling, simple particle shapes are combined with the Conical Damage Model contact law. For each type of ballast, one set of parameters is found, such that simulation and experimental results are in good accordance. A comparison with the simplified Hertz-Mindlin contact law shows several drawbacks of this model. First, the model cannot be calibrated to meet both compression and shear test results. Second, the similar behaviour in shear testing but differences in compression cannot be reproduced using the Hertz-Mindlin model. For these reasons, the CDM model is considered the better choice for the simulation of railway ballast, if simple particle shapes are used.

## Introduction

The Discrete Element Method (DEM) based modelling of railway ballast is of increasing importance. A reason is the method’s ability to gain a better understanding for phenomena occurring within the ballast bed, as for example, when new track components are introduced (e.g. elastic interlayers between sleeper and ballast). While DEM is a widely used tool for the simulation of granular materials in general, its validation for a chosen material remains a crucial point for the model’s ability of making predictions of the material behaviour.

The first step in DEM modelling is to choose a particle shape. For railway ballast several approaches can be found in the literature, which use rigid clumps of spheres, see e.g. [4, 8, 10, 13, 17], clumps of bonded spheres, see e.g. [9, 14], polyhedra, see e.g. [6, 15, 21] or so-called potential particles, see [1, 7]. Most of these works aim at a complex representation of the particles shape. This approach is always computationally demanding, no matter whether clumps of spheres, polyhedra or other particle types are used. Whether the needed effort will increase the model’s overall prediction quality will also vitally depend on the used contact law and the validation process. DEM models for railway ballast, which use simple particle shapes can also be found in the literature, see e.g [10] where box tests are considered or [4, 19] where compression and direct shear tests are considered. In [4, 10] at least qualitatively similar behaviour was found comparing DEM models with particles of different complexity.

The second step in building a DEM model is the choice of a contact law. Frequently used contact laws are the linear spring model of Cundall and Strack, [5] or the simplified Hertz-Mindlin model, see e.g. [20], which is frequently used for (clumps of) spheres. Here, in contact normal direction, the Hertz model is combined with the simplified Mindlin model together with Coulomb’s law in tangential direction. In [7], Harkness et al. conducted simulations of monotone and cyclic triaxial tests on railway ballast using potential particles. They found that the Hertz-Mindlin model was not able to fulfil the “simultaneous requirements of an apparently low initial stiffness in monotonic loading at high confining pressures (through crushing of the asperity), while retaining high elastic stiffness for the case of cyclic loading, which is of crucial importance in modelling railway ballast” [7]. To overcome this problem, they introduced a modified contact model: the Conical Damage Model (CDM). In contact normal direction the Hertz law is applied and additionally a kind of ideal plasticity is introduced to model damage at a contact. In [19] the authors of this paper faced similar problems with the Hertz-Mindlin model, in simulating compression and direct shear tests of crushed rock. These problems were solved by using a slightly modified version of the CDM model.

The parametrisation and validation of DEM models are often conducted via the comparison of simulation results with principal experiments, e.g. compression tests, direct shear tests or triaxial tests. Coetzee gives a good overview over used approaches in [4]. Often only one type of principal experiment is used. However, a DEM model validated using different types of principal experiments can be considered more trustworthy in simulating other load cases.

The current paper is a continuation of previous work. In [19], experimental data of compression and direct shear tests of crushed rock was taken from literature [4]. Using a simple particle shape (i.e. a clump of two non-overlapping equi-sized spheres), it was the aim to simulate both types of test using *one* set of parameters. It turned out that this was not possible using the simplified Hertz-Mindlin contact law. In contrast to this, using only one set of parameters the CDM model was calibrated successfully such that simulation and experimental results were in good accordance. Comparisons between experiments and simulations were conducted using all measured data instead of reducing them to single characteristics, e.g. angle of dilation. This approach is seen as a methodology for the validation of DEM models.

This paper is understood as a first part of an intended series of papers, which addresses separate parts of the DEM modelling and validation procedure and will contain the needed experimental data for two types of railway ballast. All experimental data will be made openly accessible to promote and facilitate further research on this topic. In this paper (part one), data from compression and direct shear test of the two different types of railway ballast are described in full detail together with its post-processing for the comparison with simulation results. The validation procedure developed in [19] is applied in DEM simulations using simple particle shapes. This provides further rationale that the CDM model is well suited for modelling railway ballast and its underlying physical phenomena. A comparison with the simplified Hertz-Mindlin model shows that, at least using simple particle shapes, Hertz-Mindlin has severe shortcomings.

In future steps, shape modelling will be addressed in more detail. 3d scans of the same ballast will be used to analyse the stone’s shape. The aim will be to develop particle shapes which are as simple as possible, using the gained knowledge on the stones shape. Additionally, tests on the stones’ Young’s modulus and friction test are planned to facilitate model parametrisation, which is also a challenging and usually time-consuming part of DEM model validation.

This paper is organised as follows. In Sect. 2 the experimental results on the conducted compression and direct shear tests for the two different types of ballast are described. The DEM modelling including particle shape, contact law as well as specimen generation and pre-compaction is specified in Sect. 3. The simulation results are compared to the experimental ones in Sect. 4. Finally, in Sect. 5 conclusions are drawn and an outlook on future work is given.

## Experimental results

**Fig. 1:**
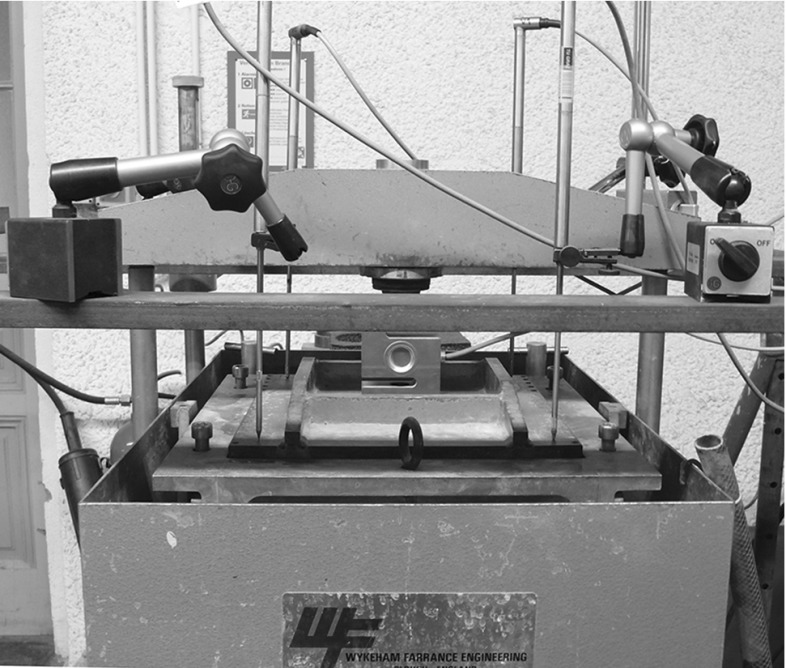
Test rig. Shown are the steel loading plate on top of the specimen, the load cell, the hydraulic load arm and the four position sensors

**Fig. 2:**
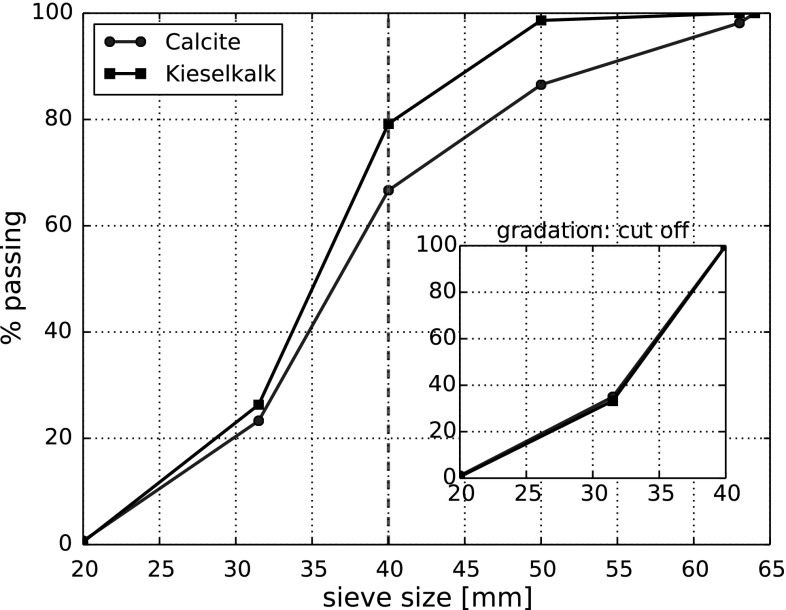
Gradation curves of the used two types of ballast. The gradation cut of at 40 mm, is shown in the included axes for both ballast types

In this work, two different types of railway ballast Calcite (stems from Croatia) and “Kieselkalk”, also known as Helvetic Siliceous Limestone, (stems from Switzerland) will be considered. Please note that the Calcite ballast does not consist of the minearal Calcite: CaCO3. The used types of ballast are two out of five types tested at Graz University of Technology at the Institute of Railway Engineering and Transport Economy in the project “LoadLabs”,^1^ see [3] (in German, abstract available in English). In this paper, compression tests as well as direct shear tests for both types of ballast will be presented. To promote further research in the field, the measured data is openly available at zenodo.org (10.5281/zenodo.1423742). Compression and direct shear test were conducted one after each other in a direct shear box test rig, see Fig. 1. The shear box itself had the size 300 mm $\times$ 300 mm $\times$ 200 mm and was divided horizontally at medium height. The size distributions of the two types of ballast are shown in Fig. 2. The maximal allowed particle size for the used test rig was 40 mm.^2^ Therefore, the gradation was cut off at this sieve size. In the inner plot of Fig. 2 it can be seen that the two types of ballast have the same size distribution, when the maximal particle size is 40 mm. Instead of cutting the particle’s size distribution, also ballast with scaled-down size distribution could have be used. In [12], it was shown that the form and shape of the smaller stones differed only slightly from the original sized ones. Further investigations on the scaled ballast were conducted in [2, 11]. Although the use of scaled down ballast would have been preferred, for this study the ballast was available only with the given size distribution. Therefore, the particle size had to be cut off.

For specimen generation, the ballast was filled in the shear box, levelled by hand and the weight of the stones was recorded. A vibrator compactor was placed on a fitting wood board and was run with 50 Hz for two minutes to compact the ballast. A steel plate was placed on top of the ballast stones and the initial height of the specimen was recorded. The median of the initial porosities was 0.447 for Calcite and 0.444 for Kieselkalk samples. Normal load was applied via a hydraulic load arm and measured by an integrated load cell in the test rig and with an external load cell. Four position sensors, which were placed near the corners of the steel plate, recorded changes in height of the specimen. This was the configuration of the test rig before the compression test as shown in Fig. 1. During both compression and direct shear test measurements of the applied normal load, the vertical path and shear path were recorded with a sampling frequency of 0.5 Hz.

### Compression tests

**Fig. 3:**
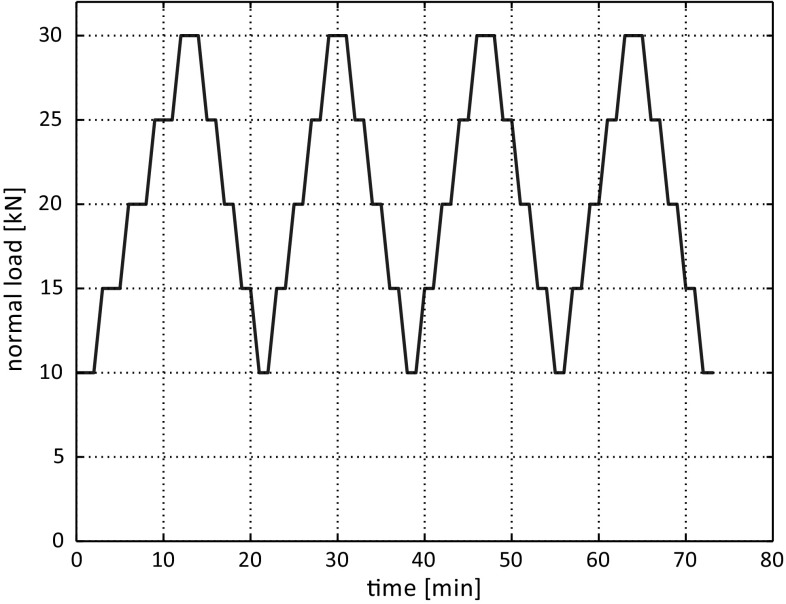
Sketch of applied load levels in compression test over time

In the compression test, five different load levels were applied: 10, 15, 20, 25 and 30 kN. These values correspond to stresses between 111 and 333 kPa. Different values of applied normal stress can be found in literature, smaller ones as well as in the same range of stresses. In this work, the applied normal load levels were chosen due to restrictions of the used test rig—lower normal loads could not be realised in a reliable way. The compression test procedure consisted of four repetitions of loading-unloading cycles using the specified normal load levels, which are sketched in Fig. 3. Each level of normal load was hold for 1 min to allow the specimen to settle. Exceptions were loading levels in the first cycle and the highest level of normal load, 30 kN, which were hold for 2 min. After the compression test, the specimens were unloaded completely. During the compression test, the normal load was controlled manually, which involved certain deviations. As several repetitions of the compression tests (always using fresh ballast) were conducted for both types of railway ballast, the results are considered reliable.

**Fig. 4:**
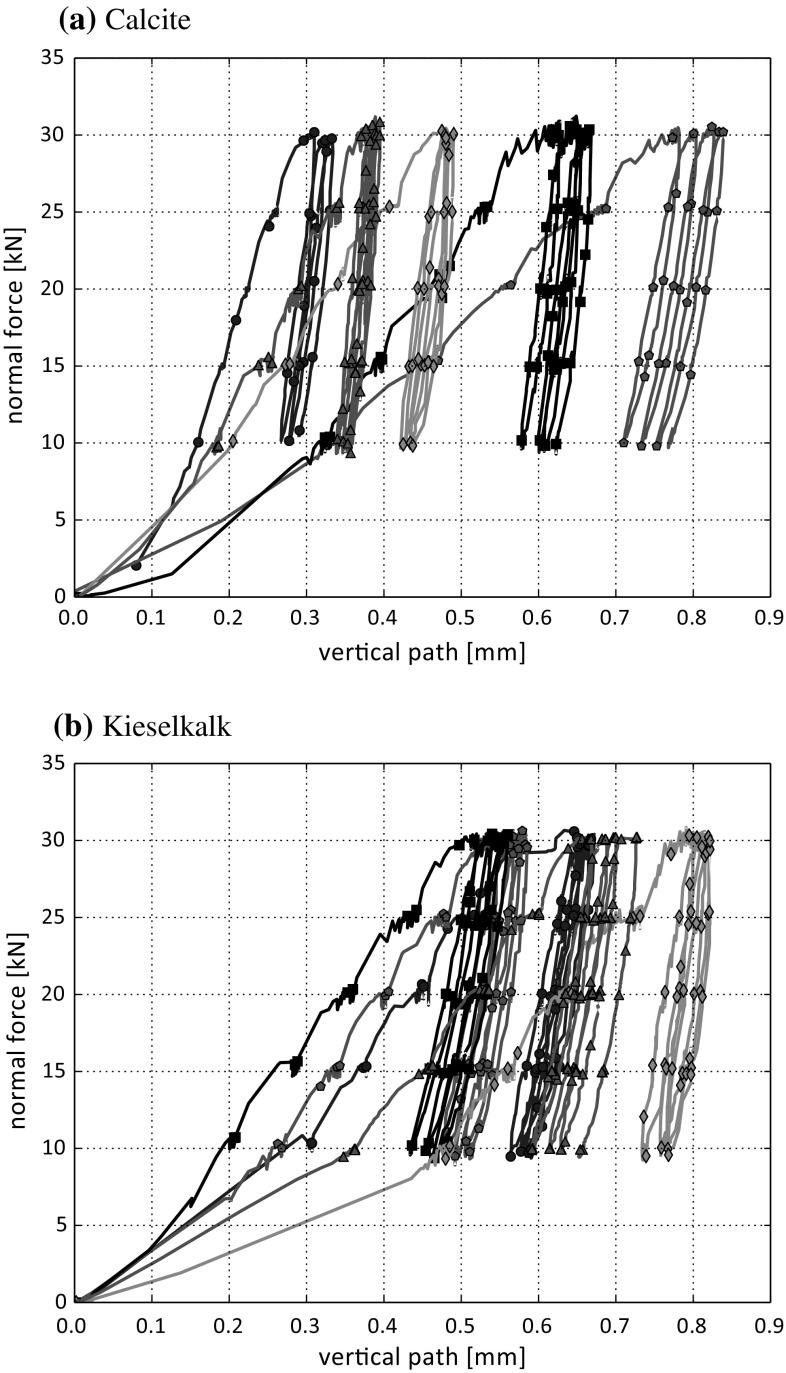
Results of five independent compression tests for the two types of ballast

In total nine compression tests were conducted for each type of ballast, Calcite and Kieselkalk. Typical results of measured normal force over vertical path can be seen in Fig. 4. Although all specimens were generated in the same way, the initial path until the first maximum in normal force is reached varies considerably for both types of ballast. This is not surprising as initially the load can be carried by only few ballast stones. Ballast re-orientation, abrasion or breakage of some load carrying stones can lead to the initial paths, which are, though smaller than 1 mm, quite large compared to the measured vertical paths during the following loading-unloading cycles. No connection between the porosity of the single specimen and the initial path can be seen from the experiments.

During loading, the slope of the curves in Fig. 4, denoted as *B*, is calculated as least-squares fit between 15 and 25 kN. When both types of ballast are compared, Kieselkalk shows less scattering in these slopes, i.e. 230–287 kN/mm, than Calcite, where the minimal slope was with 273 kN/mm considerably less steep than the maximal with 559 kN/mm.

**Fig. 5:**
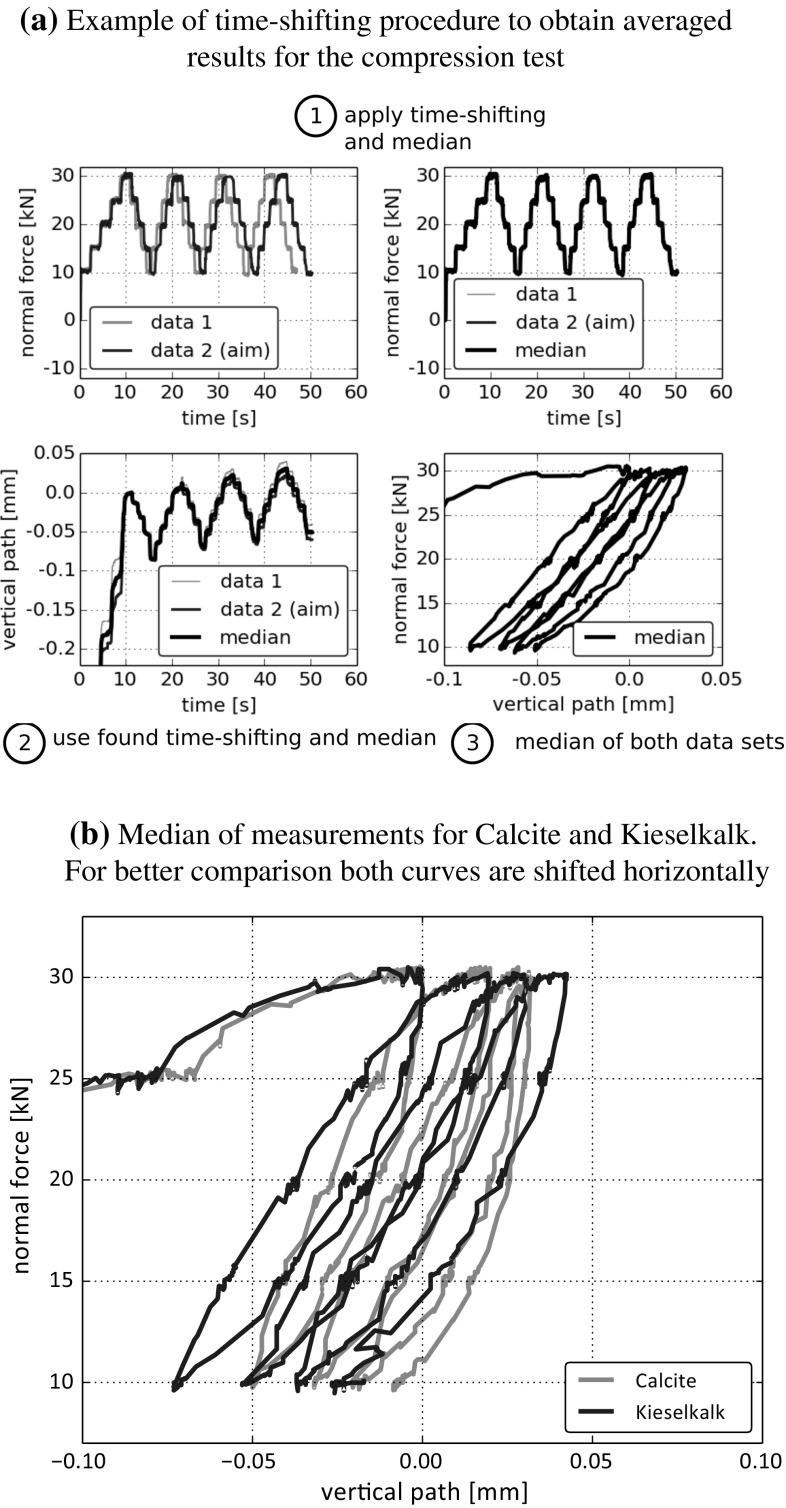
Median of the time-shifted results of compression tests for the two types of ballast

A direct comparison of the two types of ballast is not straightforward. During the tests, the normal force was controlled manually, so that the time when a certain load level was applied varied slightly. Therefore, it not possible to apply a median over all measured normal forces and vertical paths directly (for each type of ballast). To solve this problem, the measured normal forces and vertical paths are time-shifted and a so-called Dynamic Time Warping is applied (using a python open source toolbox based on [16]). An example of the utilised methodology is shown in Fig. 5a for two data sets consisting of time, normal force and vertical path each. At first, one normal force is chosen (here denoted by data 2) and the other normal force (denoted by data 1) is time-shifted to this one. The result of this time-shifting algorithm is an index rearrangement, such that data 1 is as close as possible to data 2. Now, both normal forces can be plotted over the same time measurement (belonging to data 2). The median is taken of the time-shifted measured normal forces, see Fig. 5a step 1. To time-shift the vertical path, * the same index rearrangement* is applied. In this way, the consistency of the obtained data is ensured. The median is applied to the time-shifted vertical paths in Fig. 5a step 2. Finally, in step 3 both median curves can be plotted as normal force over vertical path. Applying this procedure for several curves of Calcite and Kieselkalk separately yields the results shown in Fig. 5b. It can be seen that the slope of Calcite is with 346 kN/mm higher than the one on Kieselkalk with 272 kN/mm. The path increment (settlement) between the loading cycles is of similar magnitude for both Calcite and Kieselkalk and is with about 0.015 mm very small.

### Direct shear tests

The direct shear tests were conducted directly after the compression tests, but without the additional load cell mentioned before. For these tests, the applied normal force was measured by the integrated load cell of the test rig and again controlled manually. This configuration was chosen, because it reduced the tilt of the steel loading plate on top of the specimen, which occurred during shearing (in this way the hydraulic load arm had a bigger contact area with the steel plate). Three different values of normal load were applied 10 kN, 20 kN and 30 kN and three tests were conducted for each level of applied normal load for both types of ballast. The tests were planned with 30 mm of shear path, i.e. 10% strain, in some cases tests had to be stopped earlier due to high tilting. During shearing, the lower part of the shear box moved with 1 mm/min.

**Fig. 6:**
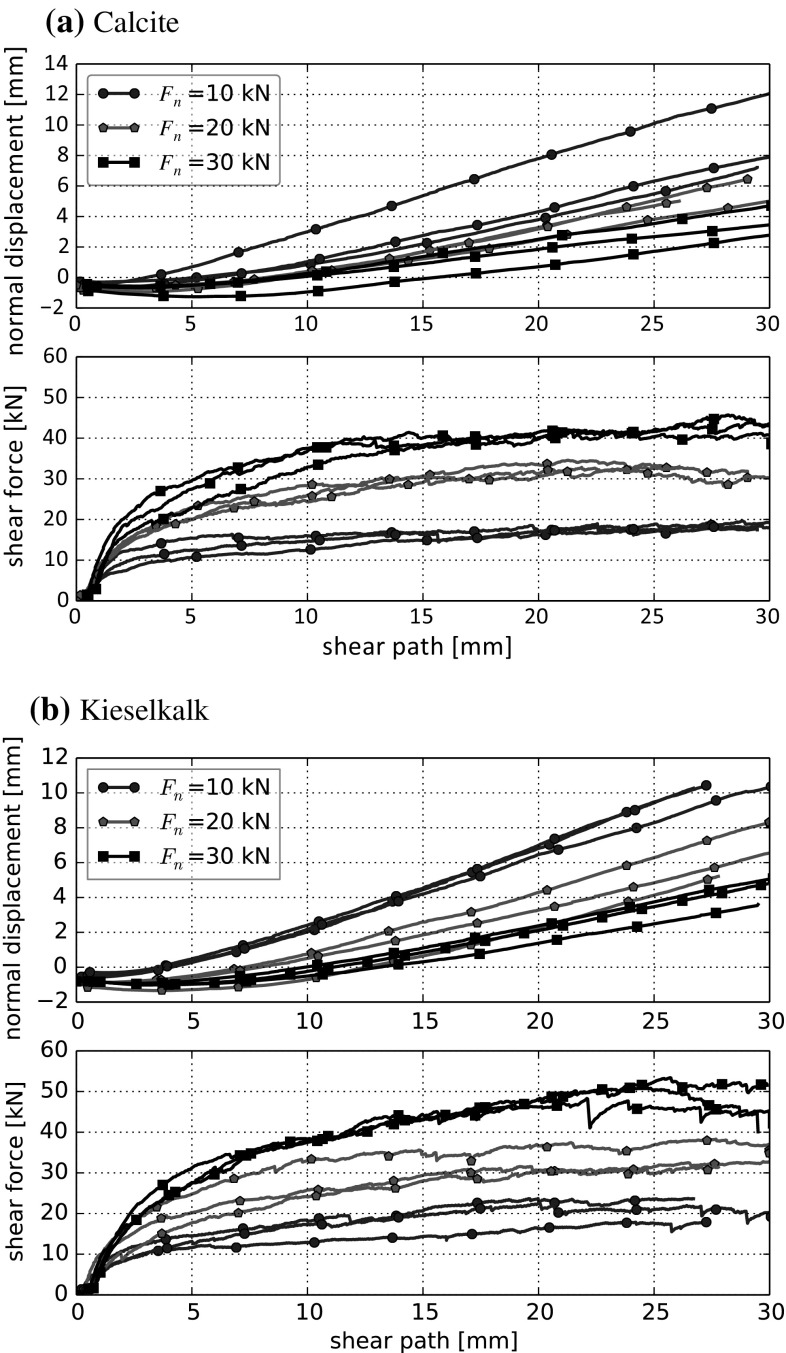
Results of direct shear tests conducted with normal loads $F_n= 10$, 20, 30 kN for the two types of ballast

**Fig. 7:**
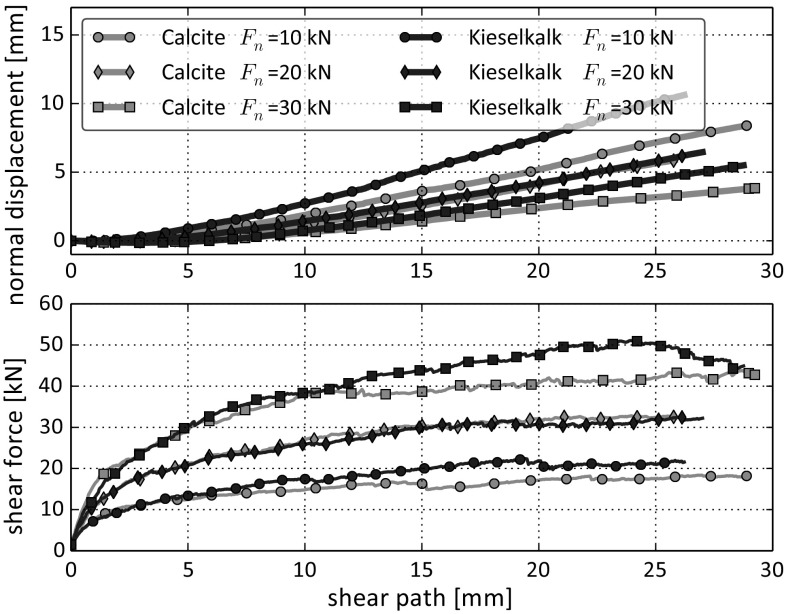
Direct comparison of direct shear tests conducted with normal loads $F_n = 10$, 20, 30 kN for the two types of ballast. Median of measurements are shown

In Fig. 6 the shear force over the shear path and the compression dilation curves are shown for both Calcite and Kieselkalk. A direct comparison of both types of ballast can be seen in Fig. 7, where the median is taken for each normal load separately. Despite the considerable differences in the behaviour of Calcite and Kieselkalk in the compression test, the results of the direct shear test are surprisingly similar. For the medium normal force, $F_n=20$ kN, the measurements are nearly identical for both types of ballast. However, for $F_n=10$ kN and 30 kN slight differences are observed, especially for shear paths larger than 15 mm. Although the specimens are rather dense with porosities around 0.46, the shear force curves do not show a clear maximum at the beginning of the test but grow monotonically. Regarding the measured normal displacement, all specimens showed a low initial contraction, followed by dilation. As expected, the lower the applied normal force the stronger is the dilation. Kieselkalk shows more dilation than Calcite, again the biggest difference is at the highest normal force.

**Fig. 8:**
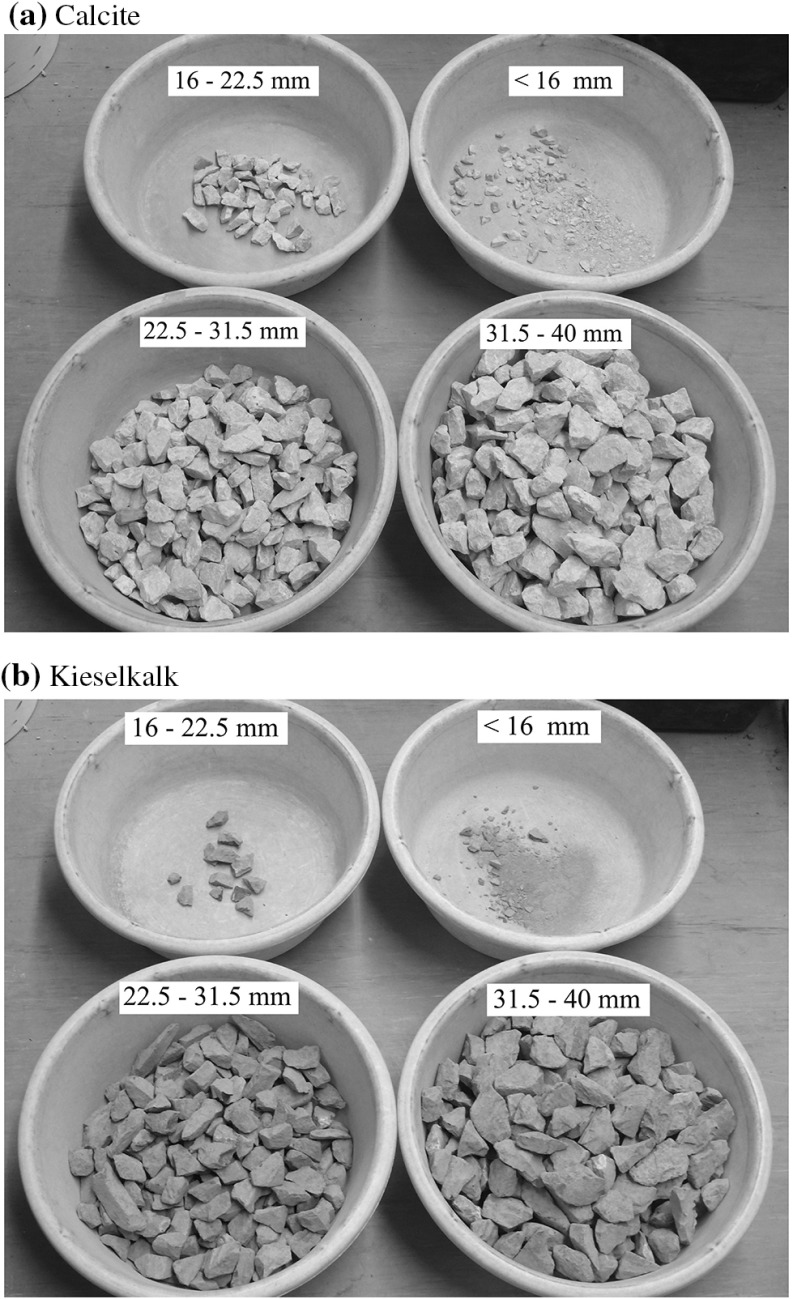
Wear and edge breakage after the after direct shear test with $F_n = 10$ kN

When the testing was finished and the ballast was removed, it could be seen that some corner breakage had occurred. Therefore, the ballast was sieved after testing for both Kieselkalk and Calcite for a test with 10 kN of applied normal load. The following sieve sizes were used: 40 mm, 31.5 mm, 22.5 mm and 16 mm, note that the initial samples contained no stones passing the 20 mm sieve. The sieved ballast is shown in Fig. 8a for Calcite and in Fig. 8b for Kieselkalk. It can be seen that some particle/edge breakage and abrasion took place, but the total extent was not too big. Comparing both types of ballast, Calcite shows a slightly higher amount of fines, thus more corner breakage/abrasion occurred, as expected.

Also, the density of the ballast was investigated with a simple water displacement method. For each type of ballast three measurements were taken.y For Calcite the median of the densities was 2822.2 kg ${\mathrm{m}^3}$ and for Kieselkalk 2660.0 kg ${\mathrm{m}^3}$.

## DEM modelling of railway ballast

### Particle geometry

High fidelity shape modelling of natural particles, such as ballast, lead to computational expensive DEM models no matter whether clumps of spheres, polyhedra or potential particles are used. Nevertheless, also in simulations with detailed shape modelling a certain degree of simplification of the reality is unavoidable. For example, effects resulting from the macroscopic particle texture cannot be considered in the particles’ shape and are thus shifted into the contact modelling (e.g. adaption of coefficient of friction).

For this reason, the current work aims to use a very simple and computationally efficient particle shape, which consists of few clumped spheres. In contrast to single spheres, the clumps can provide interlocking. The simplifications of the real particle shape and errors resulting from this simplification can be compensated, to some extent, by adapting the particle-particle contact law parameters. Clearly, this approach can only be successful, if the particle shape is not chosen too simple and the contact law incorporates all relevant physical effects. As already mentioned, in the literature simplified particle shapes are successfully employed to model railway ballast.

In a previous paper [19], the authors of this work use simple clumps of two equi-sized non-overlapping spheres to simulate the experiments of shear and compression tests presented in [4]. The experiments are conducted on samples of crushed rock and are realised in a cylindrical Jenike shear cell. Using these simple two spheres clumps good agreement with the experiments can be achieved.

**Fig. 9:**
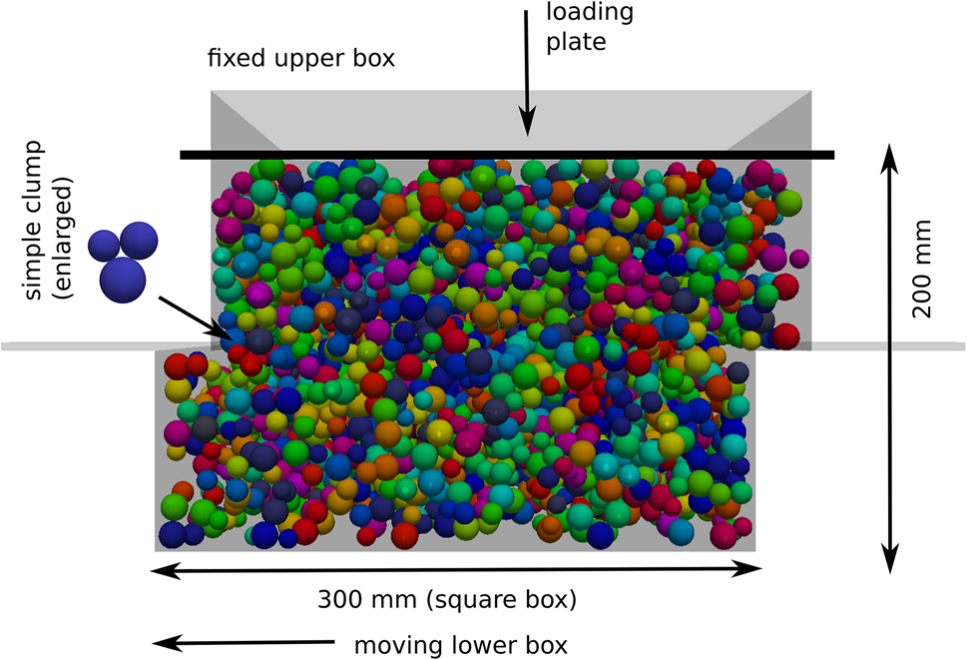
DEM model of direct shear test

In this work, for the compression and direct shear test a rectangular test rig is used. In this setup, the equi-sized two-ball clumps could lead to a so-called crystallisation (regular packings). This crystallisation was observed in simulating specimen generation for a box test using a square box of 1 m edge length (results are not part of this work). One possible solution for this problem could be to use two-ball clumps with a non-uniform particle sizes, i.e. including smaller particles. Alternatively, a different clump shape is chosen. This clump consists of three non-overlapping spheres with different radii, 7 mm, 5.8 mm and 5 mm, which are placed in a triangle. In contrast to two-spheres clumps, this arrangement of three spheres blocks rotations in all directions. This aspect is considered important for future simulations of a box test including cyclic loading. Therefore, the three-ball clump with only one clump size is preferred to a two-ball clump with non-uniform sizes (i.e. smaller particles). Regarding computational expenses, both approaches might have behaved similarly and can be considered efficient. The sides of the used test rig are 300 mm and thus the longest axis of the chosen clump is with 25.6 mm more than 10 times smaller. Fig. 9 shows the DEM model of the direct shear test, including the used simple clump shape.

### Contact law

For the DEM simulations of the compression tests and direct shear tests the software YADE [22] will be used. It is Open-Source and utilises the soft contact approach together with explicit integration in time. Due to its openness, the implementation of new or adapted contact laws is possible, whenever needed.

In [19], the authors of this work aimed to using the simplified Hertz-Mindlin contact law with the same set of parameters for both compression and direct shear tests. This turned out to be impossible (at least for the used simple clump shape), as in the Hertz-Mindlin model the Young’s modulus determines both the simulated stiffness in compression and the initial slope of the shear force leading to an unresolvable contradiction. Instead, a different contact law was applied, a modification of the conical damage model (CDM), first introduced in [7]. In the normal contact an elastic regime exists, which is modelled with Hertz law. When a certain stress is exceeded, a kind of ideal plasticity is introduced to model damage at a contact. In this phase, the surface of the DEM particle is imagined flattening locally due to damage (the geometry of the particle remains in fact unchanged). The flattening corresponds to a larger surface area and thus to a lower stress. In tangential direction, the classical Mindlin law together with a constant coefficient of friction can be used. If needed, the coefficient of friction can be adapted for every single contact to be stress dependent, as introduced in [18] or [7]. A detailed description of the CDM model equations and the used algorithm is given in [19]. With the CDM contact law the compression and shear tests could be simulated successfully with only one set of parameters. A parameter study of the CDM model allows insight in the parametrisation process and can be found in [19].

In the current work, the experimental results, which should be met by the simulations, show a surprising behaviour. The two types of ballast give very similar results in shear testing, while in the compression tests they react very differently. In simulations with one chosen particle geometry, this effect cannot be achieved with the Hertz-Mindlin contact law. Here, the similar (same) behaviour in the shear tests must result in the same behaviour in the compression tests. The only possibility to produce such an effect would be to use different particle geometries. As both types of ballast have the same gradation curve, it is doubted by the authors if the Hertz-Mindlin law can be used to simulate the described experiments. Instead, the CDM will be used, showing that the additional physical phenomena captured by this model allow for successful modelling of the considered tests. For the sake of completeness, a comparison with the Hertz-Mindlin law will also be shown.

### Specimen generation and pre-compaction

As a first step, the number of clumps of the specimen in simulations is calculated from the overall mass (from the experiments). On average 26 kg of railway ballast were used, which corresponds to simulation samples consisting of between 3300 and 3500 clumps (thus roughly 10,000 spheres). A loose cloud of clumps is generated above the shear box and is allowed to settle by gravity with a reduced interparticle friction coefficient of 0.2. In this step of the simulation, and only here, numerical damping is applied to reach the steady state faster.

In a pre-compaction step, the specimen is subjected to cyclic loading, where a constant velocity, $v^{\text {pc}}=0.05$ m/s, is assigned to the loading plate. The applied load ranges between a specified maximal force, $F^{\text {pc}}_{\text {max}}=3$ kN, and 0 and cyclic loading continues until either the target porosity, here 0.446, is met or a maximal number of cycles is reached. For the calculation of a clump’s volume, the sum of the volume of its three spheres is used (as these are not overlapping).

## Comparison with experimental results

The CDM model has five parameters, which need to be calibrated, such that the simulation results fit to the experiments. These parameters are the Young’s modulus *E*, the Poisson ratio $\nu$, the interparticle friction coefficient $\mu$ (same as in the classical Hertz-Mindlin model) and additionally a pseudo maximal compressive strength $\sigma _{\text {max}}$ and a parameter $\beta$, which relates to the part of contact overlap associated with plastic yielding. In [19], the authors showed the effect of the single parameters both on the simulated compression and direct shear tests. Using this knowledge, the parameters were fitted in a trial and error style, as computational times were too long to use an optimisation software.

A rigorous methodology for the parametrisation of this (and other DEM) material models is still missing. If parameters for a new material are sought, it might be helpful to search the literature for $E, \nu$ and $\mu$ and to set $\sigma _{\text {max}}=E/100$ and $\beta =0.01$ as a first try. As known from literature, $\mu$ is the key parameter for residual shear force and can be determined first. From the authors’ experience $\nu$ has little influence on the simulations and can be kept constant with literature values. Then, it is advised to concentrate on *E* and $\sigma _{\text {max}}$ to bring the simulated slope of the compression test and initial slope of the shear force in the direct shear test closer to their experimental counterparts. From the single parameter variation in [19] two effects are known. First, increasing *E* increases the slope in the compression test and decreases the initial slope in the direct shear test. Second, increasing $\sigma _{\text {max}}$ has the opposite effect (but to a different extent). In this fitting process, it was helpful to keep $E/\sigma _{\text {max}}$ constant, while increasing *E*. This had a considerable effect on the slope in the compression test but a much smaller effect on the direct shear test. Finally, fine tuning of the parameters is necessary to obtain the best possible agreement between simulations and measured data.

The parameters found are specified in Table 1 for both types of ballast as well as for the shear box made of steel. It is important to keep in mind that the parameter values used for the ballast should not be interpreted directly as material parameters. The simple clump shape used here is believed to influence the parameter fitting. Moreover, it is not clear, whether the available experimental data is sufficient to identify all parameters of the model uniquely. Therefore, further experiments are planned, e.g. to measure the Young’s modulus of both Calcite and Kieselkalk, which will clarify the extent of this geometrical influence on the material parameters.

**Table 1:** Material parameters used for the simulation of both types of ballast

	*E* [GPa]	$\nu [-]$	$\mu [-]$	$\sigma _{\text {max}}$ [MPa]	$\beta [-]$	$\rho$ [kg/m$^3$]
Calcite	60	0.2	0.45	600	0.0154	2822.2
Kieselkalk	30	0.2	0.45	280	0.0098	2660.0
steel box	200	0.28	0.2	–	–	7833.34

**Fig. 10:**
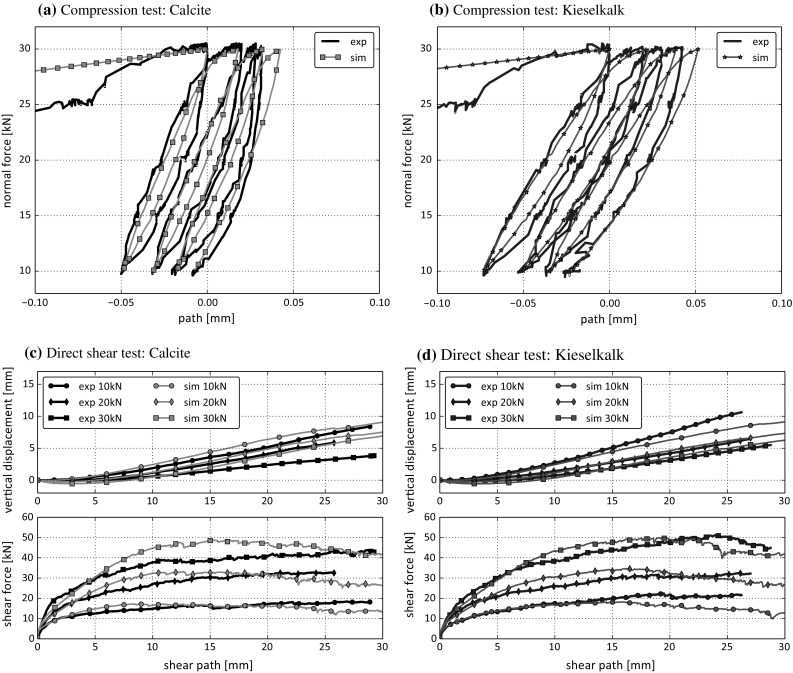
Comparison between experimental results (median) and simulation results (median) for the compression and the direct shear test using the parameter values specified in Table 1

Using the parameters in Table 1, the compression test and the direct shear test are simulated with three repetitions (i.e. initial configuration) for each test and each type of ballast. The results of the simulated compression tests are processed again with the time-shifting method before the median is applied, analogue to the experimental data in Sect. 2. For the shear test, the median is applied directly. In addition to a purely visual comparison of the results between experiment and simulation, also relative errors and curve characteristics are given, i.e. slope of compression test curve, *B*, angle of dilation, $\psi$, and secant bulk friction angle, $\phi$. The relative error between two quantities is calculated as follows:

1a $$\begin{aligned}&\varepsilon _{\text{ diff }} (a^{\text{ exp }}, a^{\text{ sim }}) =\sum _{x=0}^{x_{\text{ end }}} \Big (a^{\text{ exp }}(x) -a^{\text{ sim }}(x)\Big )^2 \end{aligned}$$

1b $$\begin{aligned}&\varepsilon _{\text{ abs }} (a^{\text{ exp }})=\sum _{x=0}^{x_{\text{ end }}} \Big ( a^{\text{ exp }}(x) \Big )^2 \end{aligned}$$

1c $$\begin{aligned}&\varepsilon _{\text{ rel }} (a^{\text{ exp }}, a^{\text{ sim }})= \sqrt{ \frac{\varepsilon _{\text{ diff }} (a^{\text{ exp }}, a^{\text{ sim }}) }{\varepsilon _{\text{ abs }} (a^{\text{ exp }}) } } \end{aligned}$$

This error is calculated in the compression test between the measured and simulated vertical path, $p^{\text{ exp }}$ and $p^{\text{ sim }}$. These quantities are again time-shifted for the calculation. In the direct shear test the relative error is calculated for the measured and simulated dilation curves, $v^{\text{ exp }}$ and $v^{\text{ sim }}$, as well as for the shear force,  and .

The obtained simulation results together with the experiments are shown in Fig. 10. The compression tests are matched very well by the simulations both for Calcite, see Fig. 10a, and for Kieselkalk, see Fig. 10b. The slopes of the simulation results are close to those of the experiments. The path increment between the load cycles, i.e. the settlement, is almost met exactly in both cases. The initial path shows a strong scattering in the experiments. Therefore, they are not compared to the simulation results. The shape of the single loading–unloading curves of the simulations is also in good accordance with the experimental results. During loading, the path-force relation is linear during most of the loading. When the applied load approaches the force maximum, the slope decreases significantly. This effect is well met by the simulation results. In Table 2 the slopes, *B*, of the experimental and simulated curves are gives together with the calculated relative errors of the vertical path. While the calculated slopes describe the good fit of only a part of the path-force curve, the low values of the relative error confirm the quality of the results over the whole range of the test.

The simulated results of the direct shear tests are also close to the experiments for both Calcite and Kieselkalk, see Fig. 10c, d. Although the simulated dilation curves are in good agreement with the experiments for both types of ballast, for Kieselkalk this is even better than for Calcite. The initial compression is also similar for both types of ballast. For Calcite all three levels of applied normal force result in more dilation than is seen in the experiments, with clear deviations for $F_n=30$ kN normal force. For Kieselkalk the dilation curves show a small deviation from the measured values only for $F_n=10$ kN at the end of the test (where also the simulated shear force drops below the measured one). In Table 3, the angles of dilation, $\psi$, and the relative errors for vertical displacement are given. To calculate $\psi$, a line was fitted through the dilation curve between 15 and 25 mm of displacement. The relatively good fit between experiment and simulation regarding the dilation curve is reflected in similar angles of dilation and low relative errors – with the two mentioned exceptions. For Calcite at $F_n=30$ kN the angle of dilation is 9.4$^\circ$ elevated and the relative error is with 0.68 quite high. On the contrary, the simulated dilation for Kieselkalk at $F_n=10$ kN is too low. Also, the angle of dilation is 7.6$^\circ$ lower than the experimental one. The relative error is with 0.18 only little higher than the value for Kieselkalk at $F_n=30$ kN. This might be confusing, when looking at the curves. The reason for this is that the absolute error, (1a), is higher in the case of $F_n=10$ kN compared to $F_n=30$ kN. But then, the absolute error is divided by the $L^2$-norm of the experimental values (1b) to obtain the relative error, resulting in similar values for both cases. Such effects need to be kept in mind, when interpreting relative errors.

The simulated shear force curve is also in good accordance with the experimental values, especially the initial part. As the shearing proceeds, the simulated shear force is slightly higher than the measured one. This effect is more pronounced the higher the applied normal force and it persists until the shear force drops below the experimental values at the end of the test. For the shear force curves the secant bulk friction angle and the relative error is given in Table 4. The secant bulk friction angle, $\phi$, is calculated for each normal load separately, assuming zero cohesion. A residual shear force is defined as the median of shear force values between 15 and 25 mm of shear path. This shear force is divided by the applied normal load and the arc tangent is taken. The secant bulk friction angle should not be confused with the bulk friction angle obtained by a regression over shear force and normal load, possibly including an apparent cohesion. For both the secant bulk friction angle and the relative error the good accordance between simulations and experiments can be seen, with slightly less agreement for the mentioned cases of Calcite at $F_n=30$ kN and Kieselkalk at $F_n=10$ kN. The differences in the secant bulk friction angle are 4$^\circ$ and 6$^\circ$, respectively. Analogously as for the dilation, the relative error is increased for Kieselkalk at $F_n=10$ kN, while this is not the case for Calcite at $F_n=30$ kN.

**Table 2:** Comparison of experimental and simulated compression test. Calculated slopes *B* of both curves and relative error of vertical path

	exp *B* [kN/mm]	sim *B* [kN/mm]	$\varepsilon _{\text{ rel }} (p^{\text{ exp }}, p^{\text{ sim }})$
Calcite	346	356	0.00015
Kieselkalk	272	244	0.00018

**Table 3:** Comparison between experimental and simulated dilation in the direct shear test using the angle of dilation $\psi$ and the relative error in vertical displacement

$F_n$	exp $\psi$	sim $\psi$	$\varepsilon _{\text{ rel }} (v^{\text{ exp }}, v^{\text{ sim }})$
Calcite			
10 kN	$20.2 ^\circ$	$20.0 ^\circ$	0.16
20 kN	$17.9 ^\circ$	$19.5 ^\circ$	0.16
30 kN	$10.0 ^\circ$	$19.4 ^\circ$	0.68
Kieselkalk			
10 kN	$26.9 ^\circ$	$19.3 ^\circ$	0.18
20 kN	$16.7 ^\circ$	$17.8 ^\circ$	0.09
30 kN	$14.9 ^\circ$	$17.3 ^\circ$	0.15

**Table 4:** Comparison between experimental and simulated shear behaviour using the secant bulk friction angle $\phi$ and the relative error in shear force

$F_n$	exp $\phi$	sim $\phi$	
Calcite			
10 kN	$59.7 ^\circ$	$58.0 ^\circ$	0.15
20 kN	$57.9 ^\circ$	$57.6 ^\circ$	0.12
30 kN	$53.5 ^\circ$	$57.5 ^\circ$	0.15
Kieselkalk			
10 kN	$64.6 ^\circ$	$58.6 ^\circ$	0.19
20 kN	$57.0 ^\circ$	$58.1 ^\circ$	0.12
30 kN	$57.8 ^\circ$	$58.3 ^\circ$	0.09

**Fig. 11:**
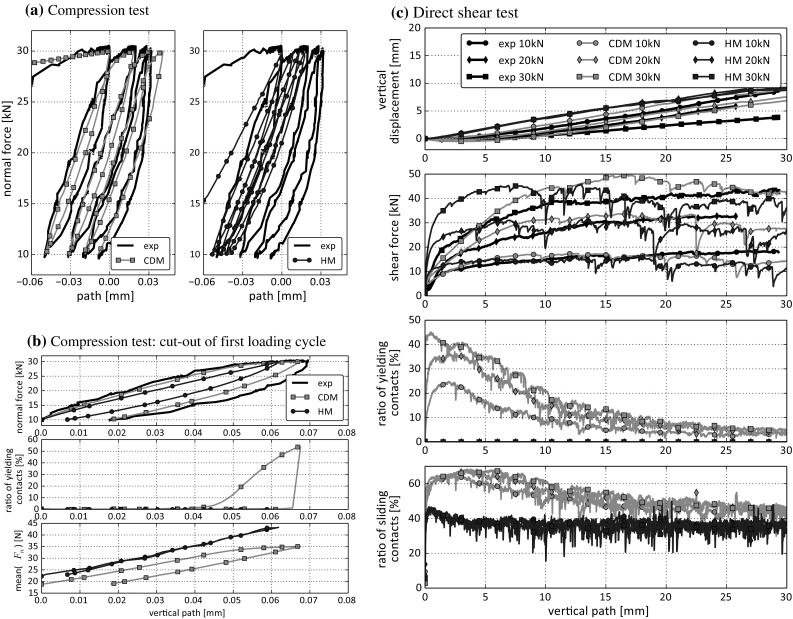
Comparison between simulations with Hertz-Mindlin and CDM model for Calcite

For completeness, a comparison between the used CDM model and the simplified Hertz-Mindlin (HM) model is conducted. Considering Calcite, a single simulation result using the CDM model is compared to one using the HM model in Fig. 11. The HM model was parametrised to fit the compression test. As it was already seen in [19] and will be seen here as well, it is not possible to parametrise the HM model such that both the compression and the direct shear tests are in good accordance with the experiments (using the simple clump shape in simulations). The parameters used for the HM model are: $$ E=150$$ GPa, $$\nu =0.2$$ and $$\mu =0.45$$. In Fig. 11a the results for the compression tests are shown. Due to parameter fitting, the slope of the HM-curves is with 321 kN/mm similar to the CDM with 356 kN/mm and the experiment with 346 kN/mm. In contrast to this, the other characteristics of the experimental path-force curve are not met well by the simulated the HM-curve. First, the path increment (settlement) between the single loading cycles is much smaller for the HM model than seen in reality. Secondly, the shape of the simulated loading cycles is in bad accordance with the experiments. In Fig. 11b this can be seen in the first subplot, where the first loading-unloading cycle is shown in cut-out. During loading the experimental curve is nearly linear until it approaches the maximum normal force: here the curve flattens. The simulation using the CDM model shows the same form, but the curve of the HM model is nearly linear over the whole range of applied normal forces. This is to be expected, as no mechanism exists in the model, which would lead to such a behaviour. The second subplot of Fig. 11b shows the ratio of the yielding contacts for the CDM model (these are identical to zero for the HM model). It is clearly seen that the flattening of the path-force curve corresponds to an increasing number of yielding contacts. This also fits to the mean normal contact force shown in the third subplot of Fig. 11b. For the HM model the mean normal contact force increases nearly linearly with the applied external normal force, while for the CDM model with yielding contacts the mean contact normal force tends towards a maximal value.

As the HM model is parametrised to fit the compression test, the agreement with experimental results of the direct shear test is poor. Dilation curves are shown in the first subplot of Fig. 11c. Here, the simulations using HM model show nearly no initial compression. Also, the results for all three levels of applied normal force coincide. The angle of dilation is somewhat lower than the experimental one. In the second subplot of Fig. 11c shear force curves are shown. The initial slopes of the results using the HM model are considerably higher than those of the experiments. Especially for $F_n=20$ kN and $F_n=30$ kN, there is a big error in shear force for shear path smaller than 10 mm. For increasing shear path, the simulation results are similar or below the experimental values. The high initial slope caused by the HM model are caused by the high Young’s modulus, which is needed to meet the results of the compression test. This is one of the main drawbacks of this model. In the CDM model the initial slopes are much closer to the experimental values. The third subplot of Fig. 11c shows the ratio of yielding contacts (which is zero for the HM model). The initial slope in the shear force of the results using the CDM model are connected to the yielding of contacts. This effect is dominant in the beginning of the test and then reduces gradually. Finally, the fourth subplot of Fig. 11c shows the ratio of sliding contacts. Here it can be seen that for increasing shear path the response of both models reaches similar sliding values and thus also similar shear force values.

## Conclusions and outlook

In this work, experimental results of compression and direct shear tests for two different types of ballast, Calcite and Kieselkalk, are presented. To promote further research in the field, the measured data is openly available at zenodo.org (10.5281/zenodo.1423742). In the experiments the behaviour of Calcite and Kieselkalk is surprisingly similar in the direct shear test, while clear differences can be seen in the stiffnesses in the compression test. A DEM model using simple clump shapes is built using the CDM contact law and one set of parameters is found for each type of ballast. The obtained simulation results are in good accordance with the experiments. This includes in the compression tests simulated stiffness, settlement and the shape of the loading-unloading cycles and in the direct shear test the shear force curve (also the initial slope) and the dilation curve. In addition to visual comparison of experimental and simulation results, error values are calculated. For completeness a simulation with the simplified Hertz-Mindlin contact law is shown for Calcite. In the DEM model using simple clumps the drawbacks of the HM model come to light. As already shown in [19], it is not possible to parametrise the HM model such that both simulated compression and direct shear tests are in good agreement with the experiments, as the Young’s modulus determines both the simulated stiffness in compression and the initial slope of the shear force. In this work, the HM model was parametrised to fit to the compression test, but still the shape of the loading-unloading curve is not met. Moreover, Calcite and Kieselkalk show similar results in the direct shear test but differ clearly in the compression test. This effect cannot be reproduced by the HM model, using the same (simple) clumps in the DEM model. It is not impossible that using different and complex shaped clumps in a DEM model could enable the HM model to show this effect. However, this is considered unlikely by the authors because yielding/edge breakage seems to be the dominant effect, which is not considered in the HM model. For all these reasons, the authors consider the CDM to be the better choice for the simulation of railway ballast, if simple clump shapes are to be used.

In future, further experiments on the same types of ballast are planned. As mentioned before, also this data will be shared openly together with the intend follow up paper. In the next step, 3D scans of ballast stones of Kieselkalk and Calcite will be conducted. Detailed shape analysis will provide information on similarities and differences between the two materials. Moreover, frictional tests and test on the Young’s modulus of both Kieselkalk and Calcite are planned. DEM shape modelling will be addressed, investigating how simple or complex the shape in simulation must be and which features can be considered in the contact model (e.g. texture). The measured material properties will also facilitate the parametrisation process. Following the open data concept, all measurements will be made openly accessible. In summary, the obtained information will be included in an improved DEM model validation methodology.

## Data Availability

The datasets generated and analysed during the current study are available in the zenodo.org repository 10.5281/zenodo.1423742.

## Footnotes

1 Project partners: Deutsche Bahn (DB), Austrian Federal Railways (ÖBB), Swiss Federal Railways (SBB) and Schweizerische Südostbahn (SOB), Institute of Railway Engineering and Transport Economy: Graz University of Technology.

2 According to the German norm DIN 18137-3:2002–09 (Baugrund, Untersuchung von Bodenproben).
